# “Easy women get it”: pre-existing stigma associated with HPV and cervical cancer in a low-resource setting prior to implementation of an HPV screen-and-treat program

**DOI:** 10.1186/s12889-023-17324-w

**Published:** 2023-12-02

**Authors:** Rachel M. Morse, Joanna Brown, Julia C. Gage, Bryn A. Prieto, Magdalena Jurczuk, Andrea Matos, Javier Vásquez Vásquez, Reyles Ríos Reátegui, Graciela Meza-Sanchez, Luis Antonio Díaz Córdova, Patti E. Gravitt, J. Kathleen Tracy, Valerie A. Paz-Soldan, the Proyecto Precancer Study Group, Iris Carhuaza, Lita E. Carrillo Jara, María del Carmen Caruhapoma, Meda Del Carpio-Morgan, Henrry Daza Grandez, Magaly Figueredo Escudero, Esther Y. Garcia Satalay, Sarah D. Gilman, Karina Gonzales Díaz, José Jerónimo, Alcedo Jorges, Anna Kohler-Smith, Margaret Kosek, Gabriela Ladrón de Guevarra, Daniel Lenin de Cuadro, Renso Lopez Liñán, Andrea Matos Orbegozo, Jaime Marín, Graciela Meza, Helen E. Noble, Victor A. Palacios, E. Jennifer Ríos López, Patricia Rivas, Karina Román, Anne F. Rositch, Carlos Santos-Ortiz, Hermann F. Silva Delgado, Sandra Soto, Nolberto Tangoa, Giannina Vásquez del Aguila, Karen Zevallos

**Affiliations:** 1 Department of Tropical Medicine and Infectious Disease, Tulane University School of Public Health and Tropical Medicine, New Orleans, LA USA; 2 Asociación Benéfica PRISMA, Lima, Peru; 3 Center for Global Health, National Cancer Institute, Bethesda, MD USA; 4 Dirección de Prevención y Control de Cancer (DPCAN) of Peruvian Ministry of Health, Lima, Peru; 5 Universidad Nacional de la Amazonía Peruana, Iquitos, Peru; 6 Hospital Regional de Loreto, Iquitos, Peru; 7 Department of Epidemiology and Public Health, University of Maryland School of Medicine, Baltimore, MD USA; 8 Department of Medicine, University of Vermont College of Medicine, Burlington, VT USA

**Keywords:** Stigma, HPV, Cervical cancer, Screening

## Abstract

**Background:**

Cervical cancer is preventable with vaccination and early detection and treatment programs. However, for these programs to work as intended, stigma related to HPV and cervical cancer must be understood and addressed. We explored pre-existing stigma associated with HPV and cervical cancer in the public healthcare system and community of a low-resource setting prior to implementation of an HPV screen-and-treat program.

**Methods:**

This study conducted thematic analysis of data collected during implementation of a novel HPV screen-and-treat system for cervical cancer early detection and treatment in Iquitos, Peru. We included 35 semi-structured interviews (19 health professionals, 16 women with cervical precancer or cancer), eight focus groups (70 community women), one workshop (14 health professionals), 210 counseling observations (with 20 nurse-midwives), and a document review. We used the Socio-Ecological Model to organize the analysis.

**Results:**

We identified three main themes: 1. the implication that women are to blame for their HPV infection through characterizations of being easy or promiscuous, 2. the implication that men are to blame for women’s HPV infections through being considered careless or unfaithful, 3. HPV is shameful, embarrassing, and something that should be hidden from others. Consequently, in some cases, women refrained from getting screened for HPV. These themes were seen at the individual level among women, relationship level among women, men, and family members, community level among healthcare staff, and societal level within components of cervical cancer guidelines and male chauvinism.

**Conclusions:**

Cervical cancer early detection and treatment programs in limited resource settings must address stigma entrenched throughout the entire healthcare system and community in order to sustainably and successfully implement and scale-up new programs. Interventions to tackle this stigma can incorporate messages about HPV infections and latency to lessen the focus on the influence of sexual behavior on HPV acquisition, and instead, promote screening and treatment as paramount preventative measures.

## Background

The large-scale elimination of cervical cancer is feasible through the implementation of human papillomavirus (HPV) vaccination for girls, screening eligible women, and providing treatment for those who need it [1, 2]. Cervical cancer early detection and treatment (EDT) programs play a crucial role in enabling health systems to reach the goal of cervical cancer elimination. These EDT programs must consider and adapt to the contexts of each health system in order to reach screening and treatment targets. One such context that challenges EDT programs is stigmatization of HPV and cervical cancer.

Stigmatization of women with HPV is a barrier to cervical cancer screening uptake [3, 4] and a barrier to completing the continuum of care following a positive screening result [5, 6]. Although most women will acquire one or more HPV infections in their lifetime that will be controlled as a benign infection [7], from women’s perspectives, HPV, abnormal Pap tests, and cervical cancer are often associated with promiscuity, guilt, self-blame, fear of social judgment, fear of rejection, and fear of discrimination by health care workers or social ties [8–11]. The stigma of having HPV has been considered by some to be more intense than a fear of having cervical cancer [10]. Literature on stigma in low- and middle-income countries (LMICs) specifically has found that women associate HPV with promiscuity, prostitution, and infidelity and commonly fear that an HPV or cervical cancer diagnosis would result in blame and rejection by their partner or social ties [6, 12, 13].

In LMICs, rates of cervical cancer exceed those of high-income countries (HICs) (18.8 vs 11.3 per 100,000 in LMICs and HICs, respectively) [14]. In the Amazonian state of Loreto in Peru, the cervical cancer mortality rate is alarmingly high at 2.6 times higher than the national average [15]. Proyecto Precancer is an implementation science project which collaborated with local stakeholders to implement a novel HPV screen-and-treat program for cervical cancer EDT in the capital of Loreto, Iquitos. The prior cervical cancer EDT program included screening for non-pregnant women aged 30–49 years with visual inspection with acetic acid (VIA) and screening for pregnant women and women aged 50–64 years with Papanicolaou (Pap) tests. All screen positive women were referred to hospital for colposcopy and appropriate treatment (e.g., cryotherapy) as needed. Determining screening coverage can be challenging in LMICs due to limited or fragmented monitoring and evaluation data [16]; however, between 2015–2017, a large survey-based study conducted with Peruvian women aged 30 years and older found that 52.4% of women had completed a Pap screening test within the last two years, and 83.2% had completed at least one Pap test in their life. Additionally, prior to implementation of the novel HPV screen-and-treat program, the same study found that 77.8% of women had heard of HPV, and 91.9% believed that HPV was the primary cause of cervical cancer [17].

We did not originally intend to explore stigma related to HPV and cervical cancer, but the theme emerged so often among women, healthcare staff, and governmental institutions during Proyecto Precancer’s work and challenged programmatic success that we decided to explore this topic retrospectively. This qualitative study explores the perspectives of various stakeholders within the EDT system, including women, nurse-midwives, doctors, and cancer coordinators on HPV and cervical cancer stigma. The data were collected during Proyecto Precancer's involvement in Iquitos, prior to implementation of the novel screen-and-treat program and include interviews, focus groups, a workshop, counseling observations, and document reviews. Our objective is to understand the existing stigma before introducing the screen-and-treat program by identifying the influence of community members, health professionals, the healthcare system, and healthcare policies in perpetuating HPV and cervical cancer stigma. This research will provide an understanding of stigma in low-resource settings that must be addressed prior to implementation of novel EDT programs to ensure appropriate and sustainable implementation.

## Methods

### Setting

This study was based in the largest public health network – the Micro Red Iquitos-Sur (MRIS) – in Iquitos, the capital city of the state of Loreto, located in the Peruvian rainforest (population 1.1 million). Iquitos is the largest city in the world that can only be accessed by boat or plane; there are no roads to Iquitos. The MRIS extends over urban, peri-urban, and rural areas, with much heterogeneity with regards to access to tertiary care. Specifically, for those in urban areas, access to a hospital could take 15 minutes on paved roads. However, for those living in peri-urban and rural areas, the main forms of transport are motorcycle taxis and boats for the river communities. The boats and motorcycle taxis frequently traveling on unpaved roads are often slow, meaning that accessing healthcare facilities, specifically tertiary care, can take hours for much of the population. Though the Amazon basin region of Peru is geographically unique from the mountain and coastal regions, this region accounts for the highest cervical cancer mortality and covers two-thirds of Peru’s territory [15].

There are approximately 33,000 women between 25–65 years old served by the MRIS’s 17 health facilities and two hospitals [18]. Some health facilities in the MRIS are small and staffed by one “*obstetra*” or nurse-midwife who is in charge of cervical cancer screening and reproductive health programs. Other facilities are large with multiple doctors and nurse-midwives. The health facilities in the MRIS serve their local communities while the hospitals serve as referral destinations for tertiary care.

### Sampling, Recruitment, and Procedures

We utilized data that had been collected throughout Proyecto Precancer’s time collaborating with stakeholders in the MRIS. Proyecto Precancer collected data throughout four phases of the INSPIRE model; however, this paper focuses solely on data collected prior to implementation of the HPV screen-and-treat program. These data were collected during INSPIRE Phases 1 and 2, and although more information on INSPIRE can be found in Gravitt et al. [19], briefly, INSPIRE Phase 1. (2017–2018) aimed to understand the previous cervical cancer EDT program (i.e., Pap or VIA screening and hospital-level follow-up), and Phase 2. (2018–2019) aimed to find leverage for changing the previous EDT program. Throughout these phases, our research team collected data through semi-structured interviews, focus groups, workshops, and document reviews with stakeholders including community women, healthcare staff, and local and national Ministry of Health staff.

The topic of stigma related to HPV and cervical cancer emerged inductively throughout Proyecto Precancer’s data collection. We then decided to retrospectively analyze a subset of the interview, focus group, and workshop data collected during Phase 1 and 2 that were relevant to stigma. The selection of these data sources was carried out by the research team, who met to identify and determine which sources were relevant to an exploration of stigma. In this article, we included 35 semi-structured interviews (19 health professionals, 16 women with cervical precancer or cancer), eight focus groups (70 community women), one workshop (14 health professionals), 210 counseling observations during VIA/Pap screening visits (20 nurse-midwives), and a document review. Table 1 summarizes each data source's participant selection, procedures, and data collection. All women (interviews, focus groups, and observed during counseling) were living in the MRIS that is made up of urban, peri-urban, and rural areas and were mestizo. All data were collected in participants’ native language, Spanish, and all participants provided written, informed consent prior to data collection.

**Table 1:** Type of data collection and description of recruitment, sample, and procedure

*Type of data collection*	*Methods*
*Semi-structured interviews* *Health professionals (n* = *19)*	***Purpose of data collection*** **:** INSPIRE Phase 1—understand the system ***Recruitment and sample***: We purposively selected key stakeholders from the MRIS working in cervical cancer EDT: eight nurse-midwives from primary healthcare facilities and 11 health professionals from the two hospitals (four doctors, three nurse-midwives, one nurse, three laboratory technicians). We approached participants about interviews at their place of work or over the phone. ***Procedure***: The interviews took place between March and October 2017, at the participant’s workplace and lasted between 30 to 60 min. The interviews used a topic guide focused on understanding how the previous VIA/Pap based cervical cancer EDT system functioned, including understanding who was involved and what information was collected during cervical cancer screening and follow-up. All interviews were conducted by J.M. (a female Peruvian biologist) and E.T. (a female American global health researcher). Interviews were audio recorded and transcribed verbatim.
*Semi-structured interviews* *Women with cervical precancer or cancer (n* = *16)*	***Purpose of data collection*** **:** INSPIRE Phase 1—understand the system ***Recruitment and sample***: We purposively selected 16 women who had received abnormal Pap screening results: cervical intraepithelial neoplasia 1, 2, or 3, or cancer in situ. Women were contacted by their primary-level nurse-midwife and invited to take part. ***Procedure***: Interviews were conducted between March 2017 and March 2018. The researcher – S.S a female Peruvian psychologist – conducted the interviews in the women's homes. The interviews lasted between 45 and 60 min and focused on women’s experiences navigating the previous VIA/Pap based EDT program including specific steps they took to receive care, and barriers and facilitators encountered throughout the process. Interviews were audio recorded and transcribed verbatim.
*Focus groups (n* = *8)* *Community women (n* = *70)*	***Purpose of data collection*** **:** INSPIRE Phase 1—understand the system ***Recruitment and sample***: We held eight focus groups in rural (n = 4) and urban (n = 4) settings in the MRIS. 70 women participated in the focus groups. Women from the rural areas were recruited through the researchers speaking to the community leader and doing a door-to-door search. Women from the urban areas were recruited while they were at the healthcare center for appointments or through a door-to-door search. All community women were eligible, regardless of whether they had previous experience with the cervical cancer EDT system. ***Procedure***: The focus groups took place between April and May 2018. In the rural settings, the focus groups were conducted in a community space, and in the urban settings were conducted in the health center. The facilitators – S.S and E.J.R.L. (a female Peruvian nurse) – used a semi-structured focus group guide exploring women’s knowledge and attitudes toward HPV prior to implementation of the HPV screen-and-treat program. Before beginning the focus groups, the facilitators provided a brief introduction to HPV, screening, and its treatment. Discussions then centered on participant’s experiences with cervical cancer screening, knowledge about HPV, and perspectives on self-sampling vs. clinician sampling. Participants also discussed three hypothetical scenarios of women who had been diagnosed with HPV: one older, married woman with three children, one middle-aged woman with a steady partner, and one young woman without a partner. The focus groups lasted between 90 and 120 min and were audio recorded and transcribed verbatim.
*Workshops (n* = *1)* *Health professionals (n* = *14)*	***Purpose of data collection*** **:** INSPIRE Phase 2—find leverage ***Sample and recruitment***: We held one “design” workshop with 14 health professionals (six nurse-midwives, one lab technician, five doctors, two regional health authorities (one cancer coordinator, one director)). We approached participants about joining the workshop at their place of work or over the phone. ***Procedure***: The workshop took place in October 2017 and lasted two full work days. It was facilitated by two primary investigators (V.P.S. a female Peruvian-American social scientist and P.E.G. a female American epidemiologist) who engaged participants in deliberative dialogues about the previous VIA/Pap based cervical cancer EDT program and evidence-based guidelines for EDT programs. The workshop focused on bidirectional knowledge exchange to clarify understandings of the cervical cancer EDT program and to find leverage for change. The workshop was audio recorded and transcribed verbatim.
*Counseling observations (n* = *210)* *Nurse-midwives (n* = *20)*	***Purpose of data collection*** **:** INSPIRE Phase 2—find leverage ***Sample and recruitment***: We conducted observations of nurse-midwives providing counseling for cervical cancer screening. Nurse-midwives were approached by the research team at their place of work. ***Procedure***: The observations took place between May and September 2019 and were conducted by G.S.S (a female Peruvian nurse-midwife) or M.J. (a female American global health researcher). The researchers observed the nurse-midwives providing information and counseling about VIA or Pap screening to either one patient (n = 209) or a group of patients (n = 1) in a private room in the healthcare facility or in a public shared community space, respectively. In the case of the one-on-one counseling sessions, the researchers took detailed notes about the observations using a semi-structured form. In the case of the group counseling, the counseling was video recorded.
*Document review*	***Purpose of data collection*** **:** INSPIRE Phase 1—understand the system and Phase 2—find leverage ***Procedure***: Our team reviewed a series of documents including: 1. Peruvian Ministry of Health cervical cancer prevention guidelines and policy documents: • The 2017–2021 National Cervical Cancer Prevention and Control Plan [20] • The 2019 Health Directive for the Prevention of Cervical Cancer [21] • The 2017 Clinical Practice Guideline for Prevention and Management of Cervical Cancer [22] 2. Regional cervical cancer screening, treatment, and follow-up forms used by the MRIS health authorities (e.g., registration forms for Pap screenings) 3. Field notes taken by researchers while collecting the data sources described above

### Data Analysis

**Fig. 1:**
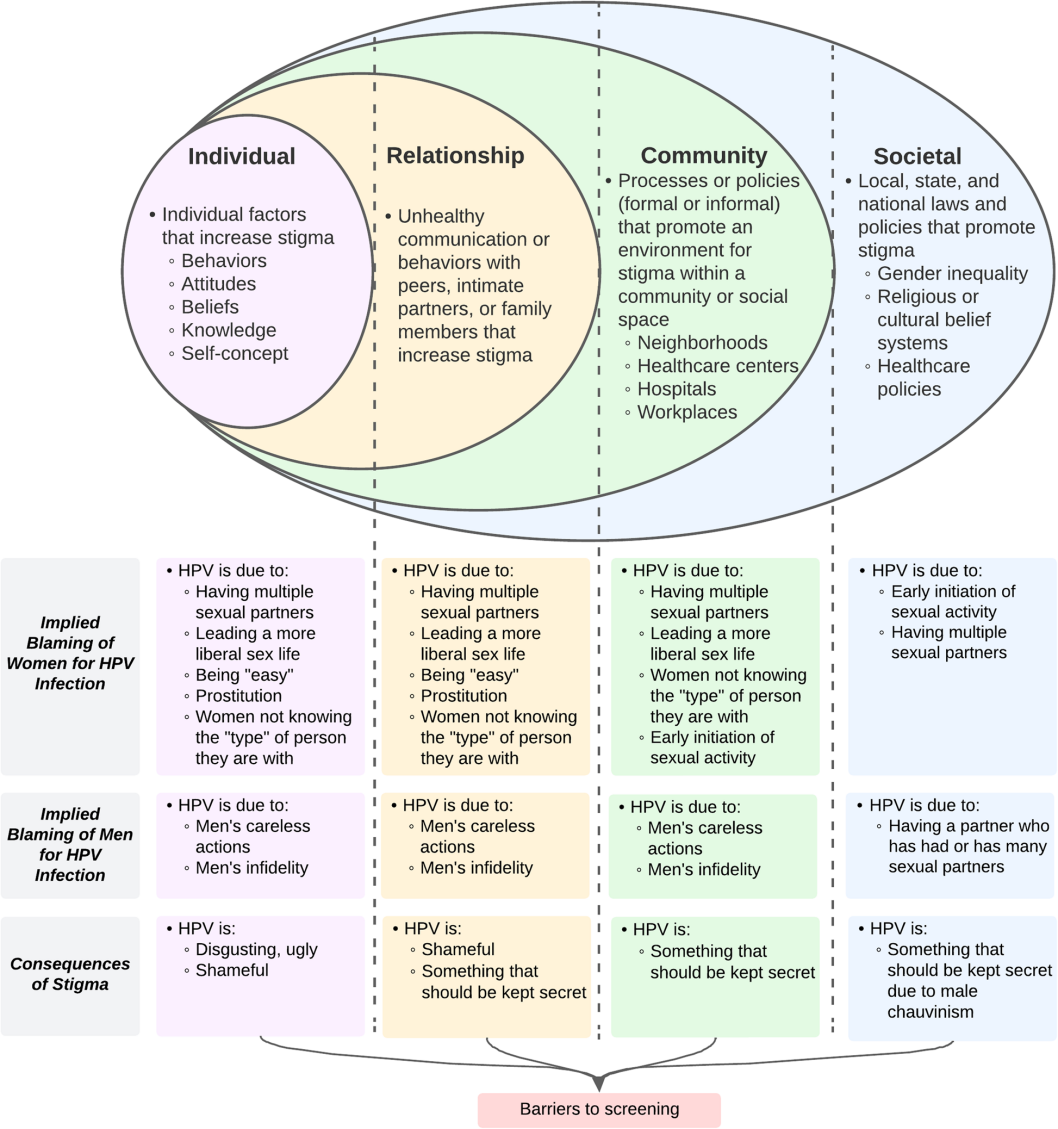
Summary of themes across health system levels prior to implementing an HPV-based screen-and-treat program

We conducted an objective-driven examination of the data sources. We first reviewed all data and subset the data into transcripts that discussed stigma and transcripts that did not. We then created a codebook based off of the Socio Ecological Model. This model, although not originally developed to examine stigma, allows consideration of how interactions among individual-level, relationship-level, community-level, and societal-level factors influence one another and perpetuate stigma [23]. We define each level of the model and the stigma that comprises it in Fig. 1. After creating the codebook, we uploaded transcripts that discussed stigma to Dedoose, and the researchers (J.B., R.M.M., and B.P.) thematically analyzed the relevant focus group, interview, and workshop transcripts in Dedoose. All transcripts were double coded, and the researchers met regularly to discuss coding and any discrepancies in the coding. Once we finished coding the transcripts, we used the codebook to conduct a manual content analysis of the documents, counseling data, and field notes.

### Ethics

The study was reviewed and approved by the ethical institutional review boards at participating institutions: Tulane University School of Public Health and Tropical Medicine (reference number 891039), the University of Maryland Baltimore (IRB#061614), Asociación Benéfica PRISMA (CE0251.09), Hospital Regional Loreto (ID-002-CIEI-2017), and Hospital Apoyo Iquitos (065-ID-ETHICS COMMITTEE HICGG- 2018).

## Results

The topic of stigma emerged inductively in interviews of eight out of 19 (42.1%) health professionals and seven out of 16 (43.8%) women. Stigma also emerged in all (8/8) focus groups, as well as the one workshop. Of the 20 nurse-midwives we observed providing counseling, four (20.0%) discussed issues related to stigma in at least one counseling session.

Overall, we found three main themes: implied blaming of women for HPV, implied blaming of men for HPV, and shame and secrecy around HPV. We summarize our themes according to the Socio-Ecological Model in Fig. 1.

### Implied Blaming of Women for HPV Infections

The implied blame of women for HPV infection emerges from comments regarding acquisition of HPV that imply women’s behavior results in their HPV. This implied blame was observed across the individual and relationship levels where community women characterized women with HPV as easy, promiscuous, or irresponsible, across the community level where nurses and nurse-midwives characterized women with HPV as sexually liberal, promiscuous, or irresponsible, and societal level where healthcare guidelines suggested a heightened importance of women’s sexual activity on HPV acquisition.

On the individual and relationship levels, women associated HPV infection with behaviors such as having multiple sexual partners and engaging in what was perceived as irresponsible sexual conduct, particularly among younger or single women. One participant spoke about how younger, sexually active women could get HPV stating, “*They experiment with boyfriends. One boy, another boy. They have relationships with different partners*” (community women's focus group #4). Another woman spoke about using the word “*pishpira”* to describe these women, “*As we say here in Loreto, we use the term ‘pishpira’. For example, she is with a man or she is with another man*” (community women's focus group #1). Women also spoke about the perception that HPV was more common among women who enjoy sex or lead a “*more liberal life*”, labeling them as “*manizers*” (invented word “*hombreriegas*”) (women's focus group #3). One woman stated, *"There are girls who like sex. They like sex and keep having sex and that ovary is not healthy. How can infections not come?"* (community women's focus group #4). Women who were more sexually liberal were also referred to as being “*easy*”, and women perceived that this sexual behavior contributed to their HPV infection. In a discussion of a scenario where a young woman finds out she has HPV, a participant described: “*She could be easy. Maybe that's why she got infected [with HPV]*” (community women's focus group #8). In some cases, having HPV was associated with prostitution. In a focus group, when the interviewer asked what participants thought of women with HPV, one woman responded: “*that they are prostitutes*” (community women's focus group #2).

Some women emphasized that it is a woman’s responsibility to know the type of person she has sex with in order to avoid HPV. One woman described this as, ***"****You have to know the person you're with, I mean, what kind of person you're messing with"* (community women's focus group #3). Other women stated that it is women’s responsibility to not have unprotected sex, even with a steady partner. During a discussion of a scenario where a woman with a partner finds out she has HPV, a participant responded: **“***We are not going to be trusting because we have a partner; it is better to be safe*” (community women's focus group #5)**.**

On the community level, these messages were similarly conveyed by health professionals. One woman reported she was told by a nurse-midwife during counseling that it is important to know the type of people she has sex with because, as the nurse-midwife told her, “*Sometimes, we don't know what kind of people we are dealing with*" (woman with precancer/cancer's interview #11). In a separate counseling session, another nurse-midwife addressed what they perceived as risky behavior and advised the patient to question her partner’s sexual history asking, “*How many partners has your partner had? Who is he?*” (nurse-midwife observation #7).

Health professionals also described women who have had multiple sexual partners as more likely to get HPV. During counseling about HPV, one nurse-midwife referred emphatically to having multiple sexual partners as an important risk factor: “*The more sexual partners we have, the more likely we are to have HPV*” (nurse-midwife observation #1). Another nurse-midwife listed risk factors for HPV during counseling as: “*promiscuity, fewer children, early initiation of sexual activity*” (nurse-midwife observation #7). In an interview, a nurse spoke about a patient with precancer as having poor sexual behavior: “*We know that she is a young woman, that she is not very well behaved*” (nurse interview #1).

A number of health professionals expressed concern that women in Iquitos are more likely to develop cervical cancer at an early age due to early initiation of sexual activity with one nurse-midwife stating, “*Here, sexual activity starts at the age of 12, 13, 14 and even more [earlier] if we talk about the communities that are [on the] rivers*” (nurse-midwife interview #6) and another stating:

*My concern is the beginning of sexual relations at an earlier age. Here we know that at 13, 14 years old they already have their first child or maybe their first partner. They arrive at 19 with more partners, even with more children. Those women are getting lost, they are going to arrive at 40 already with cancer* (workshop #1).

The discussion of women’s actions leading to their acquisition of HPV was also visible at the societal level in materials published by the Peruvian Ministry of Health. The 2017 National Clinical Practice Guidelines for Cervical Cancer Prevention and Control was created for health personnel to provide evidence-based recommendations for cervical cancer prevention. It specifies the following risk factors associated with HPV progression to cervical cancer: HPV type, genetic susceptibility (< 1% of cervical cancers), immunocompetency (nutritional status and HIV infection), tobacco use, and vitamin deficiency. Notably, a lack of screening is not mentioned as a risk factor for developing cervical cancer. Although not directly related to the development of cervical cancer, the list also mentions risk factors that significantly increase risk of HPV infection such as early initiation of sexual activity, having multiple sexual partners, and being with a partner who has multiple sexual partners [22].

The presentation of risk factors has subsequently evolved. The 2017–2021 National Plan for Prevention and Control of Cervical Cancer was created to provide guidance for public health facilities, regional and local governments, and public and private health institutions on reducing cervical cancer morbidity and mortality. It lists the factors contributing to high rates of cervical cancer: perception of population characteristics or behaviors (e.g., perceptions of sexual behavior in particular populations; in Spanish “*idiosincrasia de la poblacion*”), low screening coverage, delay in returning results, high loss to follow-up, low number of treatment centers, and concentration of specialized care in urban settings. Notably, the National Plan does not identify the same risk factors specified in the Clinical Guidelines though it was published only six months later; instead, it only lists one vague risk factor for cervical cancer related to individual sexual behavior (i.e., population characteristics or behaviors) [20].

In 2019, the Ministry of Health published the Health Directive for the Prevention of Cervical Cancer to organize services for the Ministry of Health and provide guidelines on cervical cancer prevention. Unlike the 2017 Clinical Guidelines but similar to the 2017–2021 National Plan, the absence of screening or inadequate screening appeared first on the list of factors contributing to cervical cancer, followed by HPV co-infection with Chlamydia and/or Herpes Simplex, early initiation of sexual relationships, having multiple sexual partners (two or more), having a partner who has had or has many sexual partners, not using condoms, previous or current sexual transmitted infection, multiparity (four or more pregnancies), tobacco use, and immunodeficiency [21]. Although the 2019 Health Directive prioritized the importance of screening to reduce risk of cervical cancer, risk factors associated with sexual activity remained a major focus, suggesting a continued, heightened importance of sexual activity to explain an individual’s positive screening results or diagnosis of cervical cancer, or conversely, that abstinence or safer sexual activity was required to prevent cervical screening abnormalities and cervical cancer.

Despite guidelines from the Ministry of Health on the age at which cervical cancer screening should begin, on the community level, healthcare professionals in the MRIS acted upon concerns for sexual activity risk factors and provided screening to women not yet old enough for screening but who had started engaging in sexual activity at a young age. One nurse-midwife reported, **“** *Even here in the hospital we screen all women who have initiated sexual relations within a year, because of the high risk factor that exists… because there are women who start having sexual relations at the age of 12*” (nurse interview #1). Another nurse-midwife spoke about providing all patients who are too young for screening with a VIA test, “*If it's a patient who is under the right age, I take that option of doing VIA****"*** (nurse-midwife interview #6). One nurse-midwife told women who were too young for the free screening in the SIS public health system to attend private screening instead, “*She should do the screening privately*” (nurse-midwife observation #20).

Cervical cancer screening forms used at the public, primary-level health centers for HPV, Pap, and VIA also collect data that perpetuates the association between specific sexual behaviors and HPV and cervical cancer, including age of first sexual relationship and total number of male sexual partners.

### Implied Blaming of Men for HPV Infection

The implied blame of men for women’s HPV infections was seen in discussions where men’s behaviors were implied to result in women’s HPV infections. This implied blame was observed across the individual and relationship levels among community women and women with precancer or cancer who characterized men as careless or unfaithful and the source of HPV infection for women, across the community level among cancer authorities and nurse-midwives who also characterized men as unfaithful, and across the societal level within healthcare guidelines that promoted focus on men’s sexual activity.

On the individual and relationship levels, when discussing HPV infection as a result of sexual relationships, it was common for women to first blame men’s sexual conduct for transmitting the HPV infection. Women referred to men as being careless about using protection: *“Sometimes it doesn't depend on us, and sometimes we can take care of ourselves, but sometimes men don't take care of themselves”* (woman with precancer/cancer's interview #11). Another woman stated: ***“*** *If a man has sex with a woman who is infected, he can infect as many women as he can have sex with”* (woman with precancer/cancer's interview #12).

HPV infection was also commonly viewed as a sign of male infidelity. Women spoke about what their reactions would be if they were told that they had HPV, with one woman stating, “*I would first think my partner is unfaithful to me”* (community women's focus group #5) and another woman saying, “*The man cheated*” (community women's focus group #1). Some women described feeling frustrated that, despite their efforts to care for themselves, they could still be exposed to HPV through partners they perceived to be unfaithful. One woman described that if you are living with your husband and you find out that you have HPV, the first thing you should do is speak to him because, *“He [the husband] has been promiscuous … I am paying the price"* (community women's focus group #4). Another woman described how women can develop cervical cancer due to men’s infidelity: *"Sometimes men are not faithful to you. You may think they are, but, in the end, you don't know. You can be healthy, and some time goes by, and you find out that you are sick"* (community woman's interview #11). In some cases, these women had experienced infidelity or knew women with unfaithful partners: "*My brother had sex with another woman. The woman infected him. And he went to his wife, and he infected her"* (community women's focus group #4). Only a couple of women mentioned the possibility of infection through previous relationships: *“Perhaps her partner has had other encounters before her”* (community women's focus group #8).

The implication that men are to blame for HPV infection was also observed at the community level. When asked about the risks and main ways to prevent cervical cancer, one of the cancer authorities quickly answered: “*the partners, right*?” but after thinking about it a bit more stated, “*well, but in the end, she will get it anyway*” and emphasized the importance of screening (field notes). During counseling for HPV, a nurse-midwife stated, “*Your partner infected you during sexual relations*” (nurse-midwife observation #3), and another spoke about when she used a megaphone to announce that “*faithfulness*” is one of the ways to avoid HPV (nurse-midwife interview #6). A doctor also discussed seeing a book titled *Human Papillomavirus: The Virus of Infidelity* displayed on a nurse-midwife's desk. The book was written by a nurse-midwife from Iquitos, and the doctor told this nurse-midwife, “*You can't put that [book there] because it is not entirely true. You’re going to get in trouble*” (doctor interview #2).

The implication that men are to blame for HPV infection was also observed at the societal level in the 2017 National Clinical Practice Guidelines and 2019 Health Directive for the Prevention of Cervical Cancer which suggested a heightened importance of men’s sexual activity to explain women’s positive screening results. The Clinical Practice Guidelines and Health Directive included being with a partner who has multiple sexual partners and having a partner who has had or has many sexual partners, respectively, as risk factors for progression to cervical cancer. However, the 2017–2021 National Plan does not explicitly include this risk factor related to men’s sexual behavior, and the National Plan and Health Directive both include the absence of screening or inadequate screening first on the list of factors contributing to cervical cancer [20–22].

### Consequences of Stigma—Embarrassment, Secrecy, and Lack of Screening

Women often experienced the consequences of stigma related to abnormal cervical cancer screening results and HPV. This was observed across the individual and relationship level among community women who discussed how women are told to consider and considered abnormal cervical cancer screening results to be embarrassing, in some cases disgusting, and something that should be hidden from others. This was also seen at the community level among nurse-midwives who described abnormal cervical cancer screening results as embarrassing, and the societal level where male chauvinism resulted in secrecy around HPV. As a consequence of the stigma, women sometimes refrained from getting screened for cervical cancer.

On the individual and relationship levels, women referred to HPV as “*ugly*” and felt “*disgust*” (community women's focus group #6). Similarly, women referred to stigma around gynecological symptoms they associated with abnormal cervical cancer screening results: “*Sometimes they have discharge and do not want to go to the post [because] of shame,*” (community women's focus group #6). Another woman described this as, “*Sometimes they feel the symptom and are ashamed to talk, to go to a health center*” (woman with precancer/cancer's interview #8).

Women also expressed perceived feelings of embarrassment to seek information regarding HPV or HPV screening and fears of being perceived as having HPV. During discussions of scenarios about how women with HPV might seek more information, one woman described, **“** *Maybe she doesn´t want someone to find out, so she gets information through the internet*” (community women's focus group #5) while another stated, “*I think she prefers the internet because… she could suddenly feel shame, I don’t know, to go and to ask about this virus, no?”* (community women's focus group #3).

Women were often reluctant to be seen dropping off HPV samples for fear of being judged. During a focus group, a few women discussed how if they were to do an HPV test, they would prefer to drop off the HPV samples at a secure location outside of the health center so that their neighbors would not think “*that we have cancer*” (community women's focus group #6). One of these women stated that she wanted to do this “*to keep others from seeing [her drop off the HPV test]*” (community women's focus group #6).

Women also described perceptions that their families would think differently of them if they found out they were HPV positive. During a discussion of a scenario where a young woman with HPV tells her family about her HPV infection, participants described how the family would react: **“***Because when they hear the name HPV, they would think that she is having a very liberal sex life, wouldn't they? … because the mother does not know how her daughter behaves in the street*” (community women's focus group #3).

This was also seen at the community level among nurse-midwives who support the need for secrecy; according to a nurse-midwife, some colleagues who had positive cervical cancer screening results asked not to be logged on the registry system as they preferred discreet management. Our research team also observed this, with one researcher noting that when a nurse-midwife found out about her positive result, “*the first thing she said was that she did not want to be registered in the system because she was a little ashamed*” (field notes).

Women's desire for secrecy regarding HPV was reinforced at the societal level, largely due to widespread male chauvinism. Women expressed concerns about men's ability to accept information about HPV and provide support if they became ill. During a discussion of scenarios where women with HPV tell their husbands about their HPV infections, one woman described:

*Maybe your husband sees you are sick. He is not going to want a sick woman who is suffering. Instead of supporting you, what he will do is, “You know you are sick, and I have another one out there.” He will go to the other one. He will abandon you.* (community women's focus group #4).

During a focus group, other woman discussed why women would be afraid to tell their husbands that they have HPV with one stating, “*Maybe because they are afraid of being left,”* and another adding*, “The fear of feeling rejected”* (community women's focus group #3). Another woman described how she felt a husband would react to finding out his wife has HPV: “*Suddenly, he would leave you*” (community women's focus group #1).

The stigma around HPV was described by some women as a barrier to screening:

*In order to eliminate cancer, in order to protect so many women who sometimes die because of their negligence, I say, because of their negligence, because they do not take care of themselves… sometimes they feel the symptom and are ashamed to talk, to go to a health center.* (woman with precancer/cancer's interview #8).

Male chauvinism was also mentioned as a barrier to getting screened for cervical cancer by women and health professionals. One woman spoke about why her mother had never done a Pap test before she was diagnosed with cervical cancer and died from cervical cancer a few years later:

*She said that my father always told her that, why would she go for a check-up, why would she go for a check-up if she has nothing… he was always a little bit macho in that aspect, and my mother never went for a check-up. She says that she was afraid of having a Pap smear done… I asked her, “Why are you scared?” …"I don't know, but your dad never wanted me to do that."* (woman with precancer/cancer's interview #11).

A nurse-midwife also discussed stigma related to HPV as a barrier to screening in the workshop:

*Even in our region there is a lot of male chauvinism. How many times have I visited the homes of families where the husband came out and said, “My wife is not a prostitute, she cannot have a Pap test,” even another one told me - the patient - “Look, I do not do the Pap test because I only have one husband, because they [women with HPV] have a lot of men* (workshop #1).

## Discussion

We found pervasive stigma throughout the healthcare system and community that, in some cases, led women to avoid HPV screening. This stigma may, ultimately, facilitate women dying from a preventable cancer, and subtly or overtly, it is shaped by policymakers, experts, healthcare providers and community members who have formed perceptions about who is at risk for and who might develop cervical cancer due to women’s or their partners’ sexual behaviors. Throughout this study, we found that stigma emerged unprompted in approximately half of interviews, in all focus groups, the design workshop, and among a fifth of the nurse-midwives’ providing counseling.

Stigma was associated with HPV through the implication that women are to blame for their HPV infection. This implied blame was attributed as either a direct consequence of their actions (e.g., having multiple sexual partners) or an indirect consequence (e.g., not knowing the “type” of person they are with). On the individual, relationship, and community levels, women, nurses, and nurse-midwives characterized women with HPV as promiscuous or irresponsible while on the societal level healthcare guidelines emphasized the influence of women’s sexual activity on HPV acquisition. Previous literature focusing on women’s perspectives has similarly found the implication that women are to blame for HPV infections due to having multiple sexual partners, promiscuity, prostitution, or a lack of cleanliness [9, 12, 13, 24, 25]. Additional studies have found that women often believe that HPV infection results from failure to protect oneself [9, 11, 26, 27]. Many of these previous studies, however, focus solely on the perspectives of women; our study found that this stigma is not only expressed by women but also by health professionals and within national screening guidelines.

We also found that women attributed HPV infections to men’s actions, through infidelity or carelessness. On the individual, relationship, and community levels, women, nurse-midwives, and cancer authorities characterized men as unfaithful, and on the societal level healthcare guidelines emphasized the influence of men’s sexual activity on women’s HPV acquisition. Previous studies in LMICs have found perceptions that HPV is caused by infidelity, though many of these studies discuss women being perceived as unfaithful [6, 12, 28]; however, some studies have found that HPV is perceived as associated with infidelity by both partners [24] or by men specifically [26]. We found that in addition to women implying that men are to blame for their HPV infection, healthcare workers and cervical cancer guidelines further perpetuated these concepts.

Additionally, we found attitudes of shame toward HPV. This was seen on the individual, relationship, and community levels expressed by women and nurse-midwives. The shame towards HPV, in addition to the fear of being stigmatized as being promiscuous or easy, lead both women and healthcare workers to keep their cervical cancer screening results secret. In previous research, women have described similar attitudes of perceiving HPV as shameful [24, 26] and a desire to hide their diagnosis from partners, family, and friends [26, 29]. On the individual and relationship levels, women in this study also expressed concern about rejection or abandonment by partners and families, and this was perpetuated further on the societal level with widespread male chauvinism. In some cases, this led to a reluctance to seek screening for HPV. Studies from the perspective of women have similarly found that male chauvinism is associated with stigma of HPV [30] and that women fear abandonment due to a diagnosis of cervical cancer [12]. Additional studies in LMICs have also found stigma to be a barrier to cervical cancer screening uptake [3, 4]. Our study further contributes to the literature by showing that attitudes of shame toward HPV are seen not only among community members, but also among healthcare workers.

Stigma pervades each level of the healthcare system and community, potentially intensifying as it moves through different levels. For instance, the presence of stigma among women may be exacerbated by healthcare professionals who, in turn, may have internalized stigmatized information from policies. It is important to recognize that the information flow is predominantly unidirectional; policymakers wield influence over healthcare workers and, subsequently, women, whereas women may exert limited impact on policymakers. While addressing stigma across all levels is essential, we must prioritize instigating change where it can have the greatest influence, starting with healthcare policies.

We have seen that misinterpretation and over-emphasis of less-significant risk factors related to sexual behavior stigmatizes women (regardless of whether they even have any of these risk factors). While early sexual initiation and number of sexual partners have been identified in epidemiological studies as increasing the likelihood of testing positive for HPV or developing cervical cancer [31, 32], the importance of these risk factors for individual women and public health planning is minimal compared to improving access to HPV vaccination, screening, and treatment. An HPV infection can be acquired from any sexual partner, past or present, and does not imply infidelity or promiscuity. Due to viral latency, a previous HPV infection may have gone undetected in earlier tests. Reactivation of this latent HPV infection is common [33–36], with around 43% of newly detected HPV infections attributed to reactivations of latent infections rather than new infections from recent sexual activity [35]. Any woman who has had at least one sexual relationship is at risk of acquiring HPV and therefore requires screening. Rather than fixating on the source of an HPV infection, it is vital to prioritize providing comprehensive information about HPV and its latency to women, individuals in the community, healthcare providers, and policy makers to address associations between HPV and women’s or men’s sexual behaviors. For example, in the context of HIV adherence, interventions focused exclusively on addressing stigma at the individual or relationship level yield mixed results. Such approaches overlook the role of structural factors (e.g., healthcare policies), which play a pivotal role in enhancing their overall effectiveness [37].

For HPV related stigma, this process must begin with developing national plans, directives, and clinical guidelines that consistently provide the same key messages about HPV infection and its latency and promote access to screening and treatment. Messaging on the importance of sexual activity on HPV acquisition in these documents is fallacious. The changes must include developing communication messages and cervical cancer screening forms that do not focus on or collect data on age of sexual initiation and number of male sexual partners. These materials can be developed in a participatory manner with key stakeholders, allowing them to internalize these key messages and feel ownership over new policies [38, 39]. Awareness of such policies and communication at health care facilities can protect patients through reducing discriminatory actions among healthcare providers [40].

This process must also directly target healthcare providers who play a critical role in transmitting information about and attitudes toward HPV to women. Training can aim to improve knowledge among healthcare professionals about HPV infections and promoting access to screening, while specific guidelines and tools can be developed for health professionals that help reduce misinterpretation and allow consistency in messaging. A number of interventions using these methods have been shown to reduce HIV stigma among healthcare providers [41–43] and may provide a framework for how similar programs can be developed to reduce HPV stigma among healthcare professionals.

Reducing stigma among women and community members may also include providing counseling or developing an intervention that focuses on open discussion and improving knowledge at the community and individual level. In places with high male chauvinism or that are very conservative, it might be appropriate to include men in such interventions. In fact, addressing stigma through the lens of also addressing gender equality presents an opportunity for regional authorities in low-income settings to work toward the fifth Sustainable Development Goal while providing women with further autonomy and empowerment in screening and treatment of cervical cancer [44]. Only a limited number of interventions have been developed to tackle HPV-related stigma among men and women, and their effectiveness has been mixed [45, 46]. In contrast, there are many interventions shown to address stigma associated with HIV among patients that highlight the need for HPV stigma reduction interventions to address misinformation (e.g., a focus on HPV and infidelity), humanize those with HPV (e.g., reduce association between HPV and being easy), and teach women to navigate their diagnosis [47].

During implementation of the HPV screen-and-treat program, Proyecto Precancer provided counseling and training to nurse-midwives that aimed to address stigma and improve the overall care process for women. Future research will need to evaluate the impact of this work and any further interventions implemented to address stigma.

### Limitations

This study utilizes a variety of data that were not specifically collected to examine stigma in the MRIS. As a result, it is not possible to determine the prevalence of stigma, and there is a possibility that additional stigma-related themes could emerge with further stigma-focused data collection. However, the themes and data in this paper are reflected in extensive observations made throughout years of Proyecto Precancer's fieldwork in a public health network of a low-income area in Peru and include the perspectives of multiple stakeholders, from community women to hospital-level doctors, that consistently highlight these themes. While the findings may not be generalizable to all countries or the whole country, they provide valuable information on the stigma faced by women in resource-limited settings and provide context for the scale-up and implementation of new EDT programs in these settings.

## Conclusion

Stigma is prevalent throughout this resource-limited healthcare system and community, with women, healthcare workers, and policy makers contributing to implied blame of women for their HPV infections and implied blame of men through accusations of infidelity. This stigma created feelings of shame and secrecy surrounding HPV that were present among women and nurse-midwives, and, in some cases, led women to avoid HPV screening, and ultimately, can facilitate women dying from this preventable cancer. Cervical cancer EDT programs in resource-limited settings must take into consideration and address the stigma experienced by women at the policy, health system, healthcare provider, community, and individual levels when implementing and scaling-up new EDT programs. Stigma reduction interventions should emphasize information on HPV infection and its latency over and beyond emphasis on the impact of sexual behavior on HPV acquisition to promote screening and treatment as the most important preventative measures.

## Data Availability

Data and materials can be provided on reasonable request to the corresponding author.

## Abbreviations

HPV: Human papillomavirus
EDT: Early detection and treatment
LMIC: Low- and middle-income country
HIC: High-income country
VIA: Visual inspection with acetic acid
Pap: Papanicolaou
MRIS: Micro-Red Iquitos Sur
